# Associations between parental psychopathic traits, parenting, and adolescent callous-unemotional traits

**DOI:** 10.1007/s10802-021-00841-w

**Published:** 2021-06-21

**Authors:** Hailey L. Dotterer, S. Alexandra Burt, Kelly L. Klump, Luke W. Hyde

**Affiliations:** 1 Department of Psychology, University of Michigan, Ann Arbor, MI, USA;; 2 Department of Psychology, Michigan State University, East Lansing, MI, USA; 3 Survey Research Center of the Institute for Social Research, University of Michigan, Ann Arbor, MI, USA;

**Keywords:** parenting, psychopathic traits, callous-unemotional traits, twin design, monozygotic twin differences

## Abstract

Callous-unemotional (CU) traits (i.e., callousness, low empathy, shallow affect) have been conceptualized as a downward extension of the interpersonal and affective components of adult psychopathy and are associated with stable and severe antisocial behavior. Research suggests that CU traits are moderately heritable, but also influenced by environmental factors, particularly parenting. We examined associations among mother and father psychopathic traits, parenting practices, and offspring CU traits in a community sample of 550 adolescent twins (Mean age = 13.99 years; SD 2.37; 56.4% male), incorporating multiple informants (mothers, fathers, child). Parental interpersonal-affective psychopathic traits were associated with adolescent CU traits and negative parenting (increased harshness, reduced warmth). Moreover, increased parental harshness and reduced warmth partially explained associations between parental interpersonal-affective traits and adolescent CU traits. There was also a significant direct effect specifically between mother interpersonal-affective traits and adolescent CU traits. Finally, using a twin difference design, we confirmed that adolescent CU traits were significantly impacted by non-shared environmental parenting influences (increased harshness, reduced warmth). These results suggest that mother and father interpersonal-affective traits appear to impact parenting practices and serve as risk factors for adolescent CU traits. However, many of the findings did not replicate when using cross-informant reports and were only present within single informant models, highlighting a role for shared informant variance as well. The results suggest the importance of accounting for parent personality in the development of effective parenting interventions for CU traits.

Callous-unemotional (CU) traits, including callousness, lack of empathy, and shallow affect, distinguish a distinct subgroup of youth with serious antisocial behavior (AB) (Frick et al., 2014). CU traits are associated with more stable and severe conduct problems, as well as more severe antisocial outcomes in adulthood (Frick et al., 2014). As such, understanding the development of CU traits is critical to identifying targets for intervention to prevent serious AB.

## Psychopathy and Callous-Unemotional Traits

CU traits have been conceptualized as a downward extension of traits associated with adult psychopathy, a personality construct comprised of harmful personality and behavioral features (Salekin, 2017). Given the conceptual links between CU traits and psychopathy, as well as the moderate heritability of CU traits and psychopathy (Moore et al., 2019), children with CU traits may be more likely to have parents with elevated psychopathic traits. That is, we might expect there to be associations, via heritable or familial factors, between parental psychopathy and CU traits in offspring. Importantly, psychopathy consists of distinct yet overlapping symptom sets, including interpersonal-affective features (i.e., manipulativeness, remorselessness) and impulsive-antisocial features (i.e., impulsivity, criminal versatility) (Hare & Neumann, 2008). Though CU traits are most closely related to interpersonal-affective symptoms, CU traits are also a developmental risk factor for adult AB, which overlaps with impulsive-antisocial symptoms (Frick et al., 2014). Thus, parent interpersonal-affective and/or impulsive-antisocial features could be associated with offspring CU traits.

Somewhat surprisingly, only three studies have examined whether parental psychopathic traits are associated with child CU traits, and all have done so in childhood. In community samples, Loney and colleagues (2007) found that mother psychopathic interpersonal-affective traits were associated with child CU traits (age 7–14, mean age 10 years; n=83), whereas Robinson and colleagues (2016) did not find associations between mother psychopathic traits and child CU traits (age 7–11, mean age 8 years; n=75), and neither study included fathers. In a clinical sample, Diaz and colleagues (2018) found that mother psychopathic traits (both interpersonal-affective traits and impulsive-antisocial traits) and father interpersonal-affective traits were associated with offspring CU traits (age 3 to 15, mean age 8 years; n=306). However, given that the few studies in this area have focused primarily on childhood, focus on additional developmental periods is important. For instance, numerous neurobiological, social, and cognitive changes occur in adolescence, a time when youth spend more time outside of the home and away from parents, and AB increases (Moffitt, 2018). Adolescence is also characterized by the emergence of other forms of psychopathology (Paus et al., 2008) and the stabilization of personality (Roberts & DelVecchio, 2000). Thus, it is important to clarify whether there are associations between parent psychopathy and offspring CU traits during adolescence.

In addition, developmental theory suggests that parent’s personality traits can influence parenting practices (Belsky, 1984). A previous meta-analysis found that parenting practices were broadly predicted by parent personality traits, as measured by the Big Five personality factors (Prinzie et al., 2009). Psychopathic traits are characterized by a callous and antagonistic interpersonal style (Hare & Neumann, 2008). As such, individuals high in psychopathic traits may have similarly antagonistic interactions with their children. Indeed, preliminary research suggests that psychopathic traits are related to more negative parenting (Beaver et al., 2014), less positive parenting (Schwartz et al., 2017), poor supervision (Schwartz et al., 2017), and higher levels of authoritarian parenting (i.e., low warmth and high control), as well as permissive parenting (i.e., little concern for rules or structure, place their own needs before those of the child) (Cox et al., 2018). Consistent with Belsky’s model of parenting, these parental traits may influence parenting, which may influence a cycle of dyadic interactions, described by Patterson and colleagues (1984). Through these interactions harsh parents interact with more difficult children to create coercive cycles, which, in turn result in escalating child behavior and parenting that is marked by less warmth, more harshness, and increasing inconsistency (Patterson et al., 1984). Though this research has focused on AB broadly, research indicates that parenting (harshness, low warmth) and parent-child dyadic interactions are also important in the development of CU traits (Waller & Hyde, 2017). Thus, it is possible that parental psychopathic traits may predict child CU traits indirectly via parenting practices, in addition to (or in place of) any direct (potentially heritable) effects from parental psychopathy to offspring CU traits.

Consistent with this possibility, Loney and colleagues (2007) found that negative parenting (“parenting dysfunction”) mediated the association between mother interpersonal-affective traits and CU traits in children. However, Robinson and colleagues (2016) did not find significant indirect effects from mother psychopathic traits to child CU traits. In their clinical sample, Diaz and colleagues (2018) found that specific parenting practices (i.e., negative parenting versus parental warmth) were associated with child CU traits above and beyond levels of parental psychopathy, but associations were inconsistent across informant. Researchers have posited that informant discrepancies related to parenting and family dynamics may reflect important contextual variation in children’s behavior and/or differences in informants’ perspectives of the behaviors (De Los Reyes, 2011). Moreover, previous studies have found CU traits to correlate with criterion variables differentially depending on informant (Roose et al., 2010; Thøgersen et al., 2020; White et al., 2009; Wymbs et al., 2012). Indeed, Diaz and colleagues (2018) found that mother self-reported interpersonal-affective traits were associated with father, but not mother, reported CU traits. Thus, it is important to clarify whether associations between parental psychopathy and offspring CU traits are affected by who the reporter of each construct is.

Notably, the few studies that have examined parent psychopathy, child CU traits, and parenting practices did not utilize genetically informed study designs and thus were unable to control for the effects of common genes within families. As a result, previously observed associations between parental traits, parenting, and offspring psychopathic traits may reflect gene-environment correlations (rGEs). That is, biological parents may provide both direct genetic risk (i.e., psychopathy) and environmental risk (i.e., negative parenting, low parental warmth) (passive rGE; Knafo & Jaffee, 2013). Alternatively, children at genetic risk for callous-unemotional traits that display disruptive behaviors may evoke specific parenting reactions (evocative rGE; Hawes et al., 2011; Klahr & Burt, 2014; Moore et al., 2019). One method to confirm the presence of environmental (i.e., non-genetic) transmission is examining monozygotic (MZ; identical) twin differences. By examining differences in exposure and outcomes for twins who share 100% of their DNA, researchers can determine the extent to which nonshared environmental factors influence the emergence of CU traits. Indeed, a recent cross-sectional paper in the current sample, at an earlier developmental period (in children age 6 to 11) found that twin differences in parenting practices (combined mother and father report) were related to twin differences in child CU traits. That is, the twin who experienced higher levels of harsh parenting and less parental warmth also had higher levels of CU traits (Waller et al., 2018). Additionally, Viding et al. (2009) found that differences in parent-reported negative discipline were associated with differences in parent-reported CU traits at age 7 cross-sectionally. However, differences in parent-reported negative discipline at age 7 did not predict parent-reported CU traits at age 12, controlling for earlier levels of CU traits. Thus, although nonshared environmental associations between parenting and CU traits have been found in early childhood, it is unclear whether similar environmental influences of parenting exist for *adolescent* CU traits.

Finally, developmental research has often focused specifically on the impact of mothering on child behaviors and traits. However, research suggests that there are gender differences in the expression of psychopathic traits (Efferson & Glenn, 2018). As such, the association between psychopathic traits and parenting may also differ between mothers and fathers. Previous research also suggests that there are unique associations between father versus mother psychopathic traits and behavioral phenotypes in child CU traits (Dadds et al., 2014). For example, Diaz and colleagues (2018) found differential associations among psychopathic traits, parenting, and child CU traits between mothers and fathers. However, no other studies have compared associations between mother versus father psychopathic traits, parenting, and child CU traits. Further, some research suggests etiological mechanisms of CU traits may also differ for boys versus girls (e.g., Essau et al., 2006). To this point, both Diaz and colleagues (2018) and another study in adult offspring (Auty et al., 2015) found that offspring gender significantly moderated associations among father psychopathy, parenting, and CU traits. Thus, further research is needed to examine these associations in adolescence, within a community sample including varying levels of CU traits, including multiple informants.

## Current Study

In the current study we sought to expand the literature on associations among parental psychopathic traits, parenting, and offspring CU traits in a community sample of adolescent twins that included data from both mothers and fathers. First, we examined whether parental psychopathic traits were associated with levels of adolescent CU traits. Based on findings in childhood (Diaz et al., 2018; Loney et al., 2007), we hypothesized that mother and father psychopathic traits would be directly associated with higher levels of CU traits. Second, we examined whether parental psychopathic traits were related to parenting practices. We examined measures of harshness (parental conflict) and involvement, as involvement captures a developmentally appropriate expression of warmth and engagement during late childhood and adolescence. We hypothesized that parents with higher levels of psychopathic traits would demonstrate harsher parenting and less warmth. Third, we tested whether parenting explained some of the variance in the association between psychopathic traits and child CU traits. We hypothesized that there would be indirect effects such that parental psychopathic traits would be associated with adolescent CU traits via higher levels of harshness and lower levels of warmth. Fourth, we utilized a monozygotic twin difference design to confirm whether associations between parenting and adolescent CU traits were due, at least in part, to non-shared environmental influences. We hypothesized that the twin that experienced more harsh and less warm parenting would show higher level of CU traits. In exploratory analyses, we examined whether twin gender moderated any associations. For all aims we examined whether associations differed by informant (i.e., whether findings consistent within and across informant reports).

## Methods

### Participants

Participants in this study included 550 twins (39.6% monozygotic) from 275 families living in south-central Michigan that are part of the ongoing Michigan Twin Neurogenetics Study (MTwiNS). Twins were originally recruited at age 6 – 10 for the Twin Study of Behavioral and Emotional Development in Children (TBED-C) within the Michigan State University Twin Registry (see Burt & Klump, 2019). Twins were recruited into one of two cohorts. The population-based cohort was sampled from birth records to represent all families with twins living within 120 miles of Michigan State University. The second, at-risk cohort was recruited from the same area, but only included families living in U.S. Census tracts where at least 10.5% of families lived below the poverty line (i.e., the mean for the state of Michigan at the onset of recruitment) (see Burt & Klump, 2019). The MTwiNS study was recruited from the latter subsample, as well as those in the first sample that would have qualified for the second sample (i.e., they lived in neighborhood with above mean levels of poverty), and thus represents families with twins living in neighborhoods with above average levels of family poverty. The average reported combined annual family income within MTwiNS was between $60,000 and $69,999, ranging from less than $4,999 to greater than $90,000. 12% of MTwiNS families reported an annual income below the 2017 federal poverty line of $24,600 per year and 59% reported annual income below the living wage for a family of 4 in Michigan (http://livingwage.mit.edu/states/26), consistent with a relatively low-income sample. 63 families (22.9% of families) were single parent households (i.e., only one parent figure lived in the home at time of visit). Parent-reported race was: 76.4% White/Caucasian, 14.8% Black/African American, .7% Hispanic, 1.1% Pacific Islander, 0.7% Asian, 0.7% Native American, and 5.5% Other. Participants were primarily adolescents, though the sample ranged in age from 7 to 18 years (Mean age = 13.99 years; SD 2.36; only 10.9% of the sample was 10 or younger). Notably, father self-reported psychopathic traits were only available for 206 out of the 275 families (411 child participants). These 411 participants did not significantly differ from participants without father data in mother’s education (*t*(548)=.91, *p*=.36), child gender (*x*^*2*^(1)=.01, *p*=.95), or age (*t*(548)=−1.83, *p*=.07), but did significantly differ in family annual income (*t*(539)=5.46, *p*<.001) and race (*x*^*2*^(1)=22.81, *p*<.001). Included participants with father-reported data had higher family annual income and were more likely to be White. Annual family income, and race were included as covariates in all analyses. Parents provided informed consent and children provided assent in compliance with the policies of the Institutional Review Board of the University of Michigan. Descriptives and Cronbach’s alphas for all measures are provided in Table 1.

### Measures

#### Parent psychopathic traits.

Parent psychopathic traits were assessed using the 29-item Self-Report Psychopathy Short-Form (SRP-SF; Paulhus et al., 2015). SRP-SF scores have been significantly associated with antisocial behavior in adult community samples and scores from standard interview-based assessments of psychopathy (Neumann et al., 2015). The items can be grouped into two dimensions of psychopathy: an interpersonal-affective factor (e.g., “I have pretended to be someone else in order to get something”; “I never feel guilty over hurting others”) and an impulsive-antisocial factor (e.g., “I’ve often done dangerous things just for the thrill of it”; “I have tried to hit someone with a vehicle”) (Hare & Neumann, 2008). We calculated separate summed scores of each factor for mothers and fathers. Parental psychopathy was a family-level variable (i.e., the variable is the same for both twins in the family). Though the SRP-SF can also be modeled as four facets, we opted to examine the two factors specifically to replicate previous studies (Diaz et al., 2018; Loney et al., 2007; Robinson et al., 2016) and to reduce comparisons. Moreover, the reliabilities of the facet scores were not ideal (average Cronbach’s α =.64).

#### CU traits.

CU traits were assessed using parent and child report on the 24 item Inventory of Callous-Unemotional Traits, which includes callousness (e.g., “unconcerned about feelings of others”), uncaring (e.g., “always tries best”), and unemotionality (e.g., “hides feelings”) (ICU; Essau et al., 2006; Kimonis et al., 2008). Scores from the ICU have been associated with elevated AB and reduced empathy in community samples of youth, and have been found to predict differential developmental trajectories for youth with AB (Frick et al., 2014). Consist with prior studies (Waller, Wright, et al., 2015), we calculated a separate 22-item summed scores (excluding items 10 and 23) for father-, mother-, and child-reported total adolescent CU traits.

#### Parenting.

Perceptions of parenting were assessed using parent and child report on the 42-item Parent Environment Questionnaire (PEQ; Elkins et al., 1997). Consistent with previous research (Sypher et al., 2019), we used the 12-item conflict scale to measures harsh parenting (“My parent often loses his/her temper with me”) and 12-item involvement scale to measure warm parenting (“My parent comforts me when I am discouraged or have had a disappointment”). Child report was only available on mother parenting practices. Scores on the PEQ have been associated with scale scores on other measures of family dynamics (Elkins et al., 1997) and observer ratings of parenting (Klahr et al., 2011). Additionally, the PEQ scales were associated with elevated offspring AB and CU traits in the current sample at an earlier developmental period (Waller et al., 2018). We calculated separate sum scales for self-reported (mothers and fathers) and child-reported mother conflict and involvement.

### Analytic Plan

All analyses were conducted in MPlus version 8.3 (Muthén & Muthén, 2020). For analyses in which mother-reported variables were the predictors, we used full information maximum likelihood (FIML) to accommodate missing data. However, given that it is unlikely that father-reported variables were missing at random, we did not utilize FIML in analyses in which father-reported variables were the predictors; in these cases, the sample size was restricted to those with father reports (n = 411; though with FIML within that subsample for any additional missing data). Covariance coverage ranged from 78–100% across models. To account for the nesting of siblings, all analyses were carried out using the Type=COMPLEX command (grouping variable = family ID). For all aims we examined mother versus father psychopathic traits separately. We examined a series of analyses comparing “within” informant models (i.e., same reporter for all variables) to “across” informant models (i.e., different reporters of variables). In all analyses, we controlled for parent-reported adolescent gender (0= Male, 1=Female), age, annual family income, and two parent household status (1 = two parent figures living in the home; 0 = one parent figure living in the home). We also included parent-reported adolescent race, a socially constructed category, as a covariate to control for differences in exposure to systemic racism and the unequal exposures to stress, trauma, and opportunity for people of color in the United States (0=Non-White; 1=White as White is the largest group in this sample) (Jones, 2001).

**Fig. 1.**
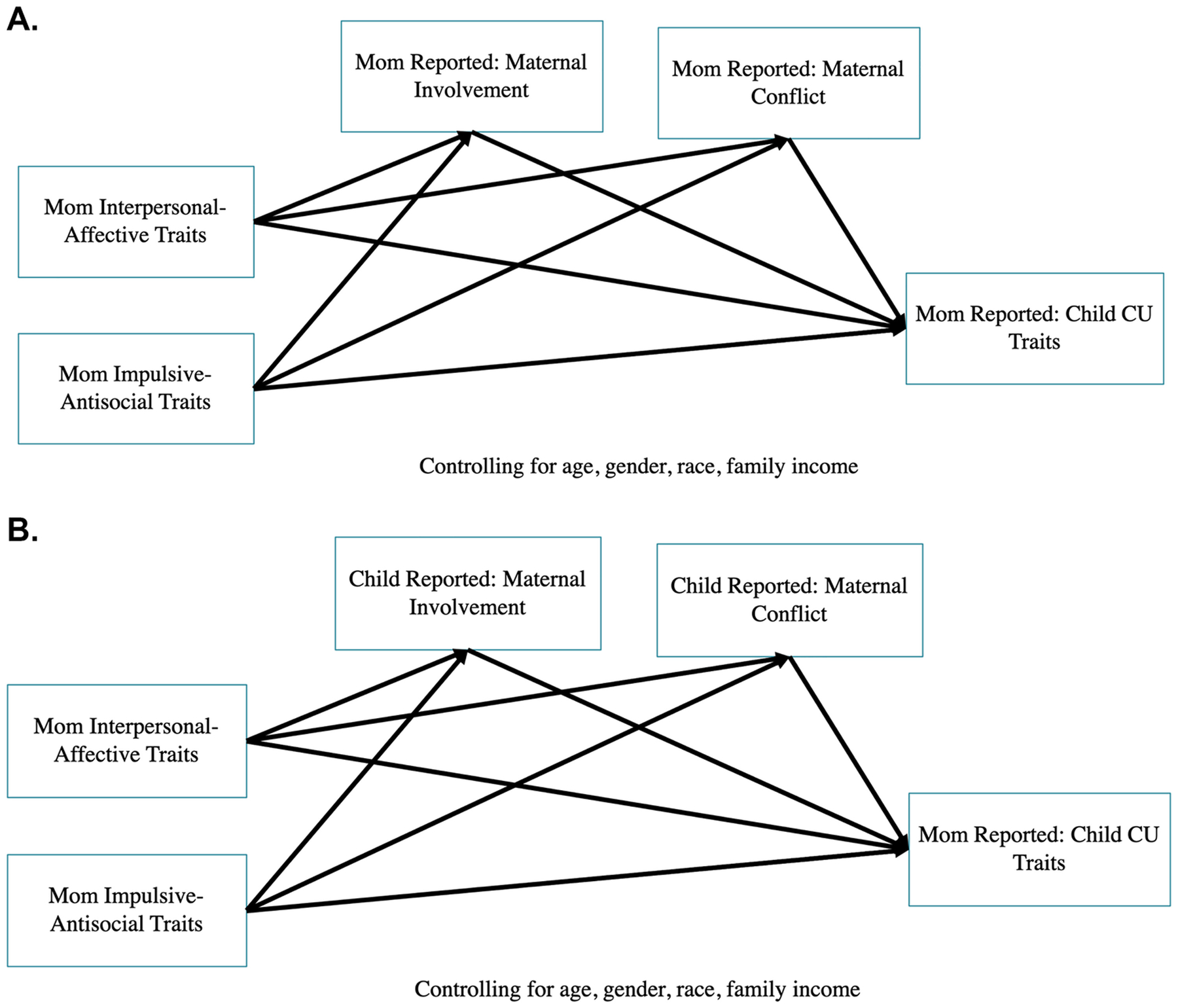
Example mediation models of associations among parental psychopathic traits, parenting, and adolescent callous-unemotional traits. In all models, parental interpersonal-affective traits and parental impulsive-antisocial traits are predictors of child callous-unemotional traits. We also modeled indirect pathways from parental interpersonal-affective traits and parental impulsive-antisocial traits to child callous-unemotional traits via parental involvement (warm/involved parenting) and parental conflict (harsh parenting). All models include child gender, race, age, two parent household status, and annual family income. Figure 1A demonstrates a “within-informant” model, such that the same informant reports on each construct within the model (i.e., mother reports on her own psychopathic traits, her own parenting, and child callous-unemotional traits). Figure 1B demonstrates an “across-informant” model, such that there are unique reporters for different constructs within the model (i.e., mother reports her own psychopathic traits and adolescent callous-unemotional traits, but child reports on mother parenting).

To address our first aim, we examined parental psychopathic traits as predictors of adolescent CU traits (see Supplemental Figure 1A–B for example models). To address our second aim, we examined parental psychopathic traits as predictors of parenting practices (parental warmth and harshness) (see Supplemental Figure 1C–D for example models). To address our third aim, we used path modeling to determine whether there were indirect effects between either of the psychopathy factors and adolescent CU traits via 1) parental harshness 2) parental warmth (four indirect paths total in each model; see Figure 1 for an example model). Parameters were estimated using ML and 95% confidence intervals (CI) for indirect effects were obtained using bias corrected bootstrapping (iterations = 5000) (Falk, 2018).

To determine whether associations between parenting and adolescent CU traits were at least partially due to non-shared environmental influences, we examined whether MZ twin differences in experiences of parenting were related to twin differences in CU traits (e.g., whether twin with higher exposure to parental harshness or less exposure to parental warmth had higher levels of CU traits). MZ twin difference analyses only included MZ twin pairs within the study, resulting in a smaller total sample size (n = 109 twin pairs). We created MZ twin difference scores for CU traits, harsh parenting, and parental warmth by subtracting Twin 2’s score from Twin 1’s score (see Table 2 for descriptives). We then examined zero-order correlations between parenting difference scores and adolescent CU traits difference scores (both across and within informant; see Supplemental Figure 2A for example models). In supplemental analyses, we also examined regressions that included difference scores for both dimensions of parenting as predictors of CU traits difference scores to determine whether associations were specific to parental warmth versus harsh parenting, consistent with previous work from this sample (Waller et al., 2018) (Supplemental Figure 2B).

Finally, to examine whether twin gender moderated any associations, we ran multi-group models for each primary aim of interest in which parameters were fixed and freed with fit compared across models using the Satorra-Bentler scaled x^2^ difference test (Satorra, 2000).

For all results, we highlight associations that met a strict conservative threshold to account for our six primary models (i.e., three primary aims, separate models for mothers and fathers) that were tested (i.e., Bonferroni-correction 0.05/6 = *p* < .008). Finally, given our focus on adolescence, all analyses were repeated excluding younger participants (i.e., 10 years of age or younger; results available upon request). The pattern of findings remained the same. Thus, we present the results using the full sample.

## Results

Zero-order correlations between adolescent CU traits and parental psychopathy as well as parenting dimensions are presented in Table 1. Within informant, higher levels of both factors of psychopathy were associated with higher levels of adolescent CU traits; however, these associations were not present across informant. Additionally, lower levels of parental warmth, and higher levels of harsh parenting were associated with adolescent CU traits. These parenting associations were significant both within and across informant (except for father-reported parenting and child-reported CU traits; Table 1).

### Are Parental Psychopathic Traits Related to Adolescent CU Traits and Parenting Practices?

First, consistent with predictions, both mother and father interpersonal-affective traits, but not impulsive-antisocial traits, were related to higher adolescent CU traits, within informant (Table 3). Both associations survived correction for multiple comparisons. There were no significant associations across informants. Second, somewhat consistent with predictions, both mother and father interpersonal-affective traits, but not impulsive-antisocial traits, were associated with reduced warmth and increased harsh parenting (Table 3). Most associations (except for father interpersonal-affective traits with parental warmth) survived correction for multiple comparisons, but were only present within informant.

### Does Parenting Explain the Association Between Parental Psychopathic Traits and Adolescent CU Traits?

Within informant, mother interpersonal-affective traits were associated with adolescent CU traits indirectly via increased harsh parenting and reduced parental warmth (Table 4; Table 5). That is, mothers higher in interpersonal-affective traits were higher in harsh parenting and lower in parental warmth, which in turn predicted higher adolescent CU traits. The direct pathway from mother interpersonal-affective traits to adolescent CU traits was also significant. These paths survived correction for multiple comparisons. There was also a significant negative direct pathway from mother impulsive-antisocial traits to adolescent CU traits, which did not survive correction for multiple comparisons.

Similarly, within informant, father interpersonal-affective traits were associated with adolescent CU traits indirectly via both parenting constructs (Table 4; Table 5). That is, fathers higher in interpersonal-affective traits were higher in harsh parenting and lower in parental warmth, which in turn predicted higher adolescent CU traits. The direct pathway from father interpersonal-affective traits to adolescent CU traits was not significant. All significant paths in the model survived correction for multiple comparisons except for one (path from father interpersonal-affective traits to father parental warmth).

Across informant, two models had either significant direct or indirect pathways. First, in the model that included mother self-reported psychopathic traits, child-reported mother parenting, and mother-reported adolescent CU traits, there was a significant direct pathway from mother interpersonal-affective traits to adolescent CU traits (Table 4; survived correction for multiple comparisons). Second, in the model that included mother self-reported psychopathic traits, mother-reported parenting, and father-reported adolescent CU traits, there was a significant indirect pathway from mother interpersonal-affective traits to father-reported adolescent CU traits via mother-reported harsh parenting (Table 5; did not survive correction for multiple comparisons).

### Are Associations Between Parenting and Adolescent CU traits At Least Partially Environmental in Origin?

Within informant, MZ twin differences in harsh parenting were significantly associated with twin differences in adolescent CU traits for all reporters (Table 2; only mother and father report survived correction for multiple comparisons). Twin differences in parental warmth were significantly associated with twin differences in adolescent CU traits for mother and child report, but not father report, though these associations did not survive correction for multiple comparisons. Across informant, twin differences in mother-reported parental warmth were associated with twin differences in father-reported adolescent CU traits, and twin differences in child-reported parental warmth were associated with twin differences in father-reported CU traits, though these associations did not survive correction for multiple comparisons. Additionally, twin differences in mother-reported harsh parenting were significantly associated with twin differences in father-reported adolescent CU traits, which survived correction for multiple comparisons. Similarly, differences in father-reported harsh parenting were associated with twin differences in mother-reported adolescent CU traits, which did not survive correction for multiple comparisons. There were no significant associations across informant when using child-reported differences in mother harsh parenting.

### Does Child Gender Moderate Associations?

We found that most associations were similar across twin gender, beyond a few exceptions (see Supplemental Materials). Only one finding survived correction for multiple comparisons. Specifically, for Aim 1, within informant, father interpersonal-affective traits were related to higher adolescent CU traits in boys (B= .36; *p*<.001), but not in girls (B= .01; *p*=.97).

## Discussion

In a community sample of twins recruited from neighborhoods with above average levels of poverty, we found that parental psychopathic traits were associated with adolescent CU traits directly and indirectly via parenting practices. Both mother and father interpersonal-affective traits were associated with higher levels of adolescent CU traits, as well as reduced parental warmth and increased harshness. Additionally, we found that both mother and father interpersonal-affective traits were associated with adolescent CU traits via reduced parental warmth with the child and increased harsh parenting. The direct effect from mother interpersonal-affective traits to adolescent CU traits remained significant when accounting for these indirect pathways. Moreover, by examining MZ differences, we confirmed that associations between parenting and CU traits were at least partially environmental in origin and not simply the result of gene-environment correlation. Taken together, parental interpersonal-affective traits may be transmitted to offspring indirectly via non-shared environmental experiences of parenting. However, many of the findings did not replicate when examining cross-informant models and were only present within single informant models, highlighting a role for shared informant variance as well. Finally, in our exploratory analyses, we found that most associations were similar across child gender, beyond one exception; however, this finding suggests that further research may be warranted to clarify the impact of child gender on pathways of transmission.

### Parental Interpersonal-Affective Traits Are Associated with Adolescent CU Traits

As hypothesized, both mother and father interpersonal-affective features were associated with higher adolescent CU traits when looking within informant. However, parental impulsive-antisocial traits were not associated with adolescent CU traits. The specificity of this association is not surprising, but important to establish, given that adolescent CU traits (e.g., lack of remorse, shallow affect) overlap more directly with the interpersonal-affective features of adult psychopathy, rather than impulsive-antisocial features (Salekin, 2017). Of note, the pattern of findings was similar for both mothers and fathers, highlighting that associations between parental psychopathy and adolescent CU traits did not differ according to parent gender. Moreover, our findings are generally consistent with previous work linking parental interpersonal-affective traits with adolescent CU traits (Diaz et al., 2018; Loney et al., 2007).

### Parental Interpersonal-Affective Traits Are Associated with Parenting Practices

As hypothesized, both mother and father interpersonal-affective features were associated with parenting practices when looking within informant. In contrast, impulsive-antisocial traits were not associated with parenting practices. This was somewhat surprising given that parent AB, which overlaps with the impulsive-antisocial traits of psychopathy, has been associated with harsher parenting (Blazei et al., 2006). However, the interpersonal-affective traits of psychopathy capture interpersonal style and social interactions more so than the impulsive-antisocial traits (Cooke & Michie, 2001), which may explain the specificity of this association, particularly in a community sample with less severe levels of AB. Overall, expanding on previous studies (Beaver et al., 2014; Cox et al., 2018; Schwartz et al., 2017), our findings suggest that parent psychopathic traits may be critical in shaping parenting practices for mothers and fathers.

### Associations Between Parental Interpersonal-Affective Traits and Adolescent Callous-Unemotional Traits Are Partially Explained by Parenting Practices

As hypothesized, there was a significant indirect pathway from parental psychopathic traits and adolescent CU traits via parenting (within informant). Specifically, consistent with previous studies (Diaz et al., 2018; Loney et al., 2007), there were significant indirect effects between both fathers’ and mothers’ interpersonal-affective traits and adolescent CU traits via reduced warmth and increased harsh parenting. Thus, one mode of transmission of parental psychopathic interpersonal-affective traits to adolescent CU traits may be via parenting, including both harsh and warm dimensions of parenting. Moreover, this indirect pathway was significant for both mothers and fathers, demonstrating further similarities in the mechanisms underlying the transmission of both mother and father psychopathic traits to adolescent CU traits. The consistency of findings across mothers and fathers is notable since we had a fairly large sample of fathers, which is rare in developmental studies (Cabrera et al., 2018). These findings are consistent with work linking coercive family processes to the development of both AB and CU traits (Patterson et al., 1984; Waller & Hyde, 2017): Parental interpersonal-affective traits may lead to harsher and less warm parenting, which contributes to coercive cycles with children, promoting the development of CU traits in youth.

Of note, mother (but not father) interpersonal-affective traits were still significantly directly associated with adolescent CU traits when including indirect pathways via parenting. The direct effect from mother psychopathic traits could reflect gender differences in expression or etiology of psychopathic traits (Efferson & Glenn, 2018; Essau et al., 2006). For example, the polygenic multiple threshold model suggests that women may require a higher liability (either due to genetic or environmental influences) to manifest antisocial behavior, given societal pressures again women expressing this behavior (Cloninger et al., 1978). Thus, women who express psychopathic traits may have a higher genetic loading, which is then transmitted to their offspring. Additionally, other unmeasured, aspects of parenting or environmental processes such as prenatal influences or neighborhood effects, could also explain this effect (Burt, 2009). Notably, previous studies have not found significant direct associations between mother psychopathic traits and adolescent CU traits when accounting for parenting (Diaz et al., 2018; Loney et al., 2007), though the current study is much larger, with greater power to identify both direct and indirect effects. Further research can clarify the sources of genetic and environmental transmission from parental psychopathic traits to child CU traits for mothers versus fathers.

### Non-shared Environmental Influences Contribute to Differences in Monozygotic Twin Callous-Unemotional Traits

Consistent with our hypotheses and a previous study using the same sample at an earlier developmental stage (childhood; Waller et al., 2018), differences in parenting between MZ twins were associated with differences in CU traits between those twins (within informant). Thus, our results emphasize that parenting continues to influence CU traits at least in part via environmental mechanisms into adolescence. However, associations with parental warmth difference scores were less robust, such that the association when using father report was only at trend-level. This finding was somewhat in contrast to Waller et al. (2018), in which parental warmth was significantly associated with CU traits using combined mother and father report. The impact of parental warmth or involvement may be greater earlier in life and less salient in adolescence, whereas harsh parenting may be more persistent across development, and thus could be a strong risk factor for CU traits across childhood and adolescence. Overall, these results provide further evidence that parenting practices are critical environmental influences on the emergence of CU traits, as has been demonstrated in previous genetically informed studies (Hyde et al., 2016; Waller et al., 2018; Waller et al., 2016; though see Viding et al., 2009). Although both CU traits (Moore et al., 2019) and parenting practices (Klahr & Burt, 2014) are somewhat heritable, taken together, our results suggest a nonshared environmental pathway from parenting to adolescent CU traits, which is not attributable to passive or evocative rGE. These results therefore highlight both the treatment potential and challenges to preventing CU traits. The association between parenting and offspring CU traits highlights parenting as a malleable target for intervention (a focus of multiple empirically supported treatments for AB). At the same time, that parents’ own psychopathic traits are associated with child CU traits and parenting, suggests that for children with CU traits, some parents may have personality traits that may be challenging for treatment providers (Viding & Pingault, 2016).

### Informant Effects

Similar to Diaz et al. (2018), we did not find associations between parental psychopathic traits and child-reported CU traits, nor did we find associations between mother psychopathic traits and child-reported parenting practices. We did find one significant cross-informant association in our MZ differences analyses that survived correction for multiple comparisons, but this was confined to cross-parent report and did not extend to child report. In fact, generally, we found little when using combinations of child and parent report across aims. These findings raise the concern that our study and others like ours, may be over-estimating the true association between parental psychopathy, parenting, and CU traits because these associations may be due, at least in part, to shared informant variance. On the other hand, these informant discrepancies could reflect important contextual variation in children’s behavior and/or differences in informants’ perspectives of the behaviors (De Los Reyes, 2011). Nevertheless, the pattern of findings was generally similar when comparing within-informant and cross-informant reports (just at a lower level of significance), arguing against informant effects as the primary explanation for our results. Moreover, previous studies utilizing observational measures of parenting have similarly identified parenting practices as an environmental factor in the development of CU traits (e.g., Hyde et al., 2016).

### Strengths and Limitations

The current study had several strengths, including the inclusion of multiple informants (mother, father, child), the examination of fathering and mothering, the examination of both parental harshness and warmth in a sample that is at greater risk for AB given the association between neighborhood poverty and AB (Ingoldsby & Shaw, 2002). Moreover, this is the first time that associations between parental psychopathy and adolescent CU traits have been examined in twins, using a genetically informed design. Despite these strengths, there are limitations worth noting. First, we examined associations in a community sample (albeit one with higher risk). Thus, our results may not be generalizable to clinical or adjudicated samples with potentially more severe levels of CU traits. Second, though it is important to examine father effects (Cabrera et al., 2018), not every family had fathers who participated in the study; as such, analyses in which father-reported variables were the predictors may have been underpowered. Children additionally did not report on their fathers’ parenting practices, and thus we were unable to examine associations between father psychopathic traits and parenting with youth reports. Third, parental psychopathy was assessed using a self-report measure. Historically, the validity of self-report measures has been questioned given that deceitfulness and manipulation are core features of psychopathy, although recent research has not found associations between psychopathic traits and response style (Ray et al., 2013). Future research incorporating multiple informants would be beneficial in further evaluating the impact of reporter perspectives on associations (De Los Reyes, 2011). Fourth, with a cross-sectional design, we were unable to determine whether earlier bidirectional associations between CU traits and parenting practices may have influenced our findings in adolescence (Hawes et al., 2011; Trentacosta et al., 2019; Waller & Hyde, 2017). Fifth, the study included a relatively wide age range; future research will be needed to clarify further associations at distinct ages. Finally, the study necessarily focused on twins, who necessarily differ from singletons (e.g., they have a sibling the same age). However, research suggests that twins and singletons are similar in the prevalence and clinical presentations of psychopathology (i.e., Pulkkinen et al., 2003); thus increasing confidence in generalization to non-twin populations.

### Conclusions

The current study found that both mother and father interpersonal-affective features were associated with parenting practices and adolescent CU traits in a community sample of twins. Moreover, the association between parental interpersonal-affective features and adolescent CU traits was partially explained by parenting practices for both mothers and fathers. We found that associations identified within informant were not robust across different informants (particularly child report). Additionally, using a genetically informed design, we demonstrated that the associations between parenting and CU traits were, at least partially, environmental. Our results provide further evidence that 1) CU traits are not entirely attributable to genetic risk and 2) that parenting significantly impacts child outcomes via environmental mechanisms, while also demonstrating that parent personality can influence parenting practices. The results are consistent with models of the determinants of parenting that suggest that parent personality factors contribute to parenting style, which, in turn, influence parent-child interaction and child outcomes (Belsky, 1984; Waller, Shaw, et al., 2015). Thus, considering both parent personality and parenting practices are likely critical to designing effective intervention strategies targeting CU traits (Viding & Pingault, 2016).

## Supplementary Material

1732839_Sup

## Figures and Tables

**Table 1:** Descriptives and Zero-Order Correlations Between Parental Psychopathy, Adolescent Callous-Unemotional Traits, and Dimensions of Parenting

	Descriptives				Zero-Order Correlations		
	*n*	*M(SD)*	Range	α	Adolescent CU Traits (Mom Report)	Adolescent CU Traits (Dad Report)	Adolescent CU Traits (Child Report)
Mother Interpersonal-Affective Traits	506	18.85(5.36)	13–39	.81	.26***	.08	.07
Mother Impulsive-Antisocial Traits	506	17.28(3.67)	14–29	.67	.11*	.00	.07
Father Interpersonal-Affective Traits	411	22.66(7.01)	14–42	.83	.09^+^	.22***	.03
Father Impulsive-Antisocial Traits	416	19.85(5.51)	13–42	.76	.06	.17**	.05
Mother Involvement (Mom Report)	477	43.26(4.12)	27–48	.79	−.54***	−.25***	−.22***
Mother Conflict (Mom Report)	477	20.20(5.91)	11–43	.88	.51***	.30***	.17***
Mother Involvement (Child Report)	471	40.36(6.14)	15–48	.89	−.31***	−.30***	−.47***
Mother Conflict (Child Report)	470	20.81(6.76)	11–47	.87	.26***	.22***	.30***
Father Involvement (Dad Report)	335	40.69(5.48)	16–48	.88	−.18**	−.52***	−.08
Father Conflict (Dad Report)	337	20.10(5.81)	12–41	.88	.21***	.54***	.08
Adolescent CU Traits (Child Report)	524	17.97(6.82)	1–45	.77	.39***	.36***	
Adolescent CU Traits (Dad Report)	392	17.41(8.44)	0–52	.87	.50***		
Adolescent CU Traits (Mom Report)	545	16.42(8.65)	0–45	.87			

Note.

+ *p* < .10,

* *p* < .05,

** *p* < .01,

*** *p* < .001.

CU = callous-unemotional. M = mean. SD = standard deviation. Conflict = scale of harsh parenting. Involvement = scale of warm parenting.

**Table 2:** Descriptive Statistics Intra-Class Correlations for Study Variables Computed to Establish Associations Within Monozygotic (MZ) Twin Pairs, Including 95% CI, and Correlations Between Monozygotic Twin Difference Scores of Adolescent Callous-Unemotional Traits and Dimensions of Parenting

	Descriptives			Intra-Class Correlations	Twin Difference Score Correlations		
	*n*	Twin 1 *M (SD)*	Twin 2 *M (SD)*	Total *r* (95% CI)	Adolescent CU Traits (Mom Report)	Adolescent CU Traits (Dad Report)	Adolescent CU Traits (Child Report)
Adolescent CU Traits (Mom Report)	108	15.86(8.43)	14.94 (8.45)	.44*** (28–.58)			
Adolescent CU Traits (Dad Report)	77	17.10 (8.20)	16.73 (8.11)	.70*** (.57–.80)	.49***^1^		
Adolescent CU Traits (Child Report)	101	18.44 (7.00)	17.97 (6.99)	.50*** (.34–.63)	.24*	.23*	
Mother Involvement (Mom Report)	94	43.78 (3.99)	43.46 (4.21)	.70*** (.59–79)	−.31**^1^	−.29*	−.09
Mother Conflict (Mom Report)	94	20.05 (6.25)	19.77 (6.11)	.70*** (.59–.79	.35**^1^	.41***^1^	.15
Mother Involvement (Child Report)	92	40.05 (6.60)	39.20 (7.20)	.68*** (.55–.77)	−.20^+^	−.26*	−.26*
Mother Conflict (Child Report)	91	22.18 (7.79)	21.30 (7.37)	.70*** (.58–.79)	.13	.23^+^	.26*
Father Involvement (Dad Report)	67	41.00 (5.15)	41.09 (5.51)	.86*** (.78–.91)	−.15	−.24^+^	−.16
Father Conflict (Dad Report)	67	20.31 (5.42)	19.24 (5.38)	.70*** (.55–.80)	.25*	.53***^1^	.05

Note.

+ *p* < .10,

* *p*<.05,

** *p*<.01,

*** *p*<.001.

1 = survived for multiple comparisons (.05/6 = p < .008).

CU = callous-unemotional. M = mean. SD = standard deviation. There were 109 monozygotic twin pairs out of 275 total twin pairs. Smaller *ns* represent missing data. Conflict = scale of harsh parenting. Involvement = scale of warm parenting.

**Table 3:** Associations Among Parental Psychopathic Traits, Parenting, and Adolescent CU Traits

Associations Between Parental Psychopathic Traits and Adolescent CU Traits												
	Adolescent CU Traits (Mom Report)				Adolescent CU Traits (Dad Report)				Adolescent CU Traits (Child Report)			
df = 23	B	SE	*p*	R^2^	B	SE	*p*	R^2^	B	SE	*p*	R^2^
**Mothers**												
Mother Interpersonal-Affective Traits	.30^1^	.06	<.001		.13	.10	.18		.04	.06	.45	
Mother Impulsive-Antisocial Traits	−.09	.06	.15	.15***	−.07	.09	.41	.04	.00	.06	.96	.13***
df = 14	B	SE	*p*	R^2^	B	SE	*p*	R^2^	B	SE	*p*	R^2^
**Fathers**												
Father Interpersonal-Affective Traits	.12	.08	.13		.23^1^	.09	.007		.02	.09	.81	
Father Impulsive-Antisocial Traits	−.04	.08	.59	.11*	−.01	.09	.91	.08^+^	−.02	.09	.84	.14**

Associations Between Parental Psychopathic Traits and Dimensions of Parenting																
	Mother Involvement (Mom Report)				Mother Conflict (Mom Report)				Mother Involvement (Child Report)				Mother Conflict (Child Report)			
df = 33	B	SE	*p*	R^2^	B	SE	*p*	R^2^	B	SE	*p*	R^2^	B	SE	*p*	R^2^
**Mothers**																
Mother Interpersonal-Affective Traits	−.23^1^	.07	.001		.22^1^	.07	.003		−.05	.07	.43		.12	.07	.11	
Mother Impulsive-Antisocial Traits	−.01	.07	.91	.14**	.07	.07	.39	.12**	−.03	.07	.70	.07^+^	−.04	.07	.60	.08*
	Father Involvement (Dad Report)				Father Conflict (Dad Report)											
df = 24	B	SE	*p*	R^2^	B	SE	*p*	R^2^								
**Fathers**																
Father Interpersonal-Affective Traits	−.25	.11	.02		.31^1^	.09	.001									
Father Impulsive-Antisocial Traits	.11	.11	.33	.09	.02	.10	.87	.13*								

Note.

1 = survived for multiple comparisons (.05/6 = *p* < .008).

+ *p*<.10,

* *p*<.05,

** *p*<.01,

*** *p*<.001.

CU = callous-unemotional. B = standardized regression weight. df = degrees of freedom. All models included parent-reported adolescent gender, race, age, two parent household status, and annual family income. Models also included both factors as predictors to account for their overlap. In a separate set of analyses, all models were also repeated without covariates and the pattern of results was the same with one exception such that mother interpersonal-affective traits were also significantly associated with child-reported mother harsh parenting (*p*= .03). Conflict = scale of harsh parenting. Involvement = scale of warm parenting.

**Table 4:** Path Coefficients and Direct Effects for Models of Parental Psychopathic Traits, Parenting, and Adolescent CU Traits

Within Informant Model 1: Mother-Reported Parenting and Adolescent CU Traits	Mother Involvement (Mom Report)				Mother Conflict (Mom Report)				Adolescent CU Traits (Mom Report)			
df = 43	B	SE	*p*	R^2^	B	SE	*p*	R^2^	B	SE	*p*	R^2^
Mother Interpersonal-Affective Traits	−.24^1^	.07	.001		.23^1^	.07	.002		.16^1^	.06	.003	
Mother Impulsive-Antisocial Traits	−.01	.07	.92	.14**	.06	.08	.45	.12**	−.11	.05	.02	.35***
Mother Involvement (Mom Report)									−.35^1^	.06	.00	
Mother Conflict (Mom Report)									.31^1^	.05	.00	
Within Informant Model 2: Father-Reported Parenting and Adolescent CU Traits	Father Involvement (Dad Report)				Father Conflict (Dad Report)				Adolescent CU Traits (Dad Report)			
df = 34	B	SE	*p*	R^2^	B	SE	*p*	R^2^	B	SE	*p*	R^2^
Father Interpersonal-Affective Traits	−.26	.10	.01		.31^1^	.09	.001		.05	.08	.56	
Father Impulsive-Antisocial Traits	.12	.11	.24	.08	.001	.10	.99	.12*	.03	.08	.73	.33***
Father Involvement (Dad Report)									−.35^1^	.08	<.001	
Father Conflict (Dad Report)									.37^1^	.07	<.001	
Across Informant Model 1: Child-Reported Parenting and Mother-Reported Adolescent CU Traits	Mother Involvement (Child Report)				Mother Conflict (Child Report)				Adolescent CU Traits (Mom Report)			
df = 43	B	SE	*p*	R^2^	B	SE	*p*	R^2^	B	SE	*p*	R^2^
Mother Interpersonal-Affective Traits	−.05	.07	.42		.11	.07	.13		.29^1^	.06	<.001	
Mother Impulsive-Antisocial Traits	−.03	.08	.68	.07^+^	−.04	.07	.62	.08*	−.09	.06	.13	.20***
Mother Involvement (Child Report)									−.21	.06	<.001	
Mother Conflict (Child Report)									.07	.06	.20	
Across Informant Model 2: Mother-Reported Parenting and Father-Reported Adolescent CU Traits	Mother Involvement (Mom Report)				Mother Conflict (Mom Report)				Adolescent CU Traits (Dad Report)			
df = 43	B	SE	*p*	R^2^	B	SE	*p*	R^2^	B	SE	*p*	R^2^
Mother Interpersonal-Affective	−.24^1^	.07	.001		.23^1^	.08	.002		.05	.10	.63	
Mother Impulsive-Antisocial	−.01	.07	.90	.14**	.06	.08	.46	.12**	−.09	.09	.29	.11^+^
Mother Involvement (Mom Report)									−.10	.08	.20	
Maternal Conflict (Mom Report)									.25^1^	.06	<.001	

Note.

1 = survived for multiple comparisons (.05/6 = *p* < .008).

+ *p*<.10,

* *p*<.05,

** *p*<.01,

*** *p*<.001;

CU = callous-unemotional. B = standardized regression weight. df = degrees of freedom. All models included parent-reported adolescent gender, race, age, two parent household status, and annual family income. Models also included both factors as predictors to account for their overlap. In a separate set of analyses, all models were also repeated without covariates and the pattern of results was the same. Conflict = scale of harsh parenting. Involvement = scale of warm parenting.

**Table 5:** Total and Indirect Effects for Models of Parental Psychopathic Traits, Parenting, and Adolescent CU Traits

Within Informant Model 1: Mother-Reported Parenting and CU Traits	Estimate			Bias-Corrected Bootstrap 95% CI	Non-Corrected Bootstrap 95% CI
	B	SE	*p*		
Total Interpersonal-Affective Traits	.32^1^	.06	<.001	.19, .44	.19, .44
Interpersonal-Affective 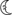 Involvement 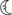 CU Traits	.09^1^	.03	.002	.04, .15	.03, .14
Interpersonal-Affective 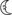 Conflict 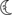 CU Traits	.07^1^	.03	.007	.03, .13	.02, .13
Total Impulsive-Antisocial Traits	−.09	.06	.15	−.21, .03	−.21, .03
Impulsive-Antisocial 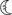 Involvement 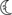 CU Traits	.002	.02	.92	−.04, .05	−.04, .05
Impulsive-Antisocial 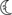 Conflict 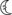 CU Traits	.02	.02	.46	−.03, .07	−.03, .07
Within Informant Model 2: Father-Reported Parenting and CU Traits	Estimate			Bias-Corrected Bootstrap 95% CI	Non-Corrected Bootstrap 95% CI
	B	SE	*p*		
Total Interpersonal-Affective Traits	.25^1^	.09	.005	.08, .42	.08, .43
Interpersonal-Affective 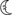 Involvement 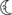 CU Traits	.09	.04	.03	.02, .19	.02, .18
Interpersonal-Affective 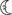 Conflict 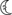 CU Traits	.11^1^	.04	.006	.05, .21	.04, .20
Total Impulsive-Antisocial Traits	−.02	.09	.86	−.19, .16	−.19, .16
Impulsive-Antisocial 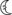 Involvement 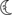 CU Traits	−.04	.04	.26	−.13, .02	−.12, .03
Impulsive-Antisocial 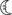 Conflict 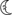 CU Traits	.00	.04	.99	−.07, .07	−.07, .07
Across Informant Model 1: Child-Reported Parenting & Mother-ReportedCU Traits	Estimate			Bias-Corrected Bootstrap 95% CI	Non-Corrected Bootstrap 95% CI
	B	SE	*p*		
Total Interpersonal-Affective Traits	.31	.06	.00	.18, .43	.18, .42
Interpersonal-Affective 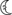 Involvement 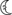 CU Traits	.01	.02	.44	−.02, .04	−.02, .04
Interpersonal-Affective 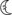 Conflict 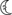 CU Traits	.01	.01	.38	−.003, .04	−.02, .03
Total Impulsive-Antisocial Traits	−.09	.06	.15	−.21, .03	−.20, .04
Impulsive-Antisocial 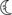 Involvement 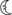 CU Traits	.01	.02	.70	−.02, .05	−.02, .05
Impulsive-Antisocial 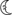 Conflict 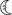 CU Traits	−.00	.01	.72	−.03, .01	−.02, .01
Across Informant Model 2: Mother-Reported Parenting and Father-Reported Adolescent CU Traits	Estimate			Bias-Corrected Bootstrap 95% CI	Non-Corrected Bootstrap 95% CI
	B	SE	*p*		
Total Interpersonal-Affective Traits	.13	.10	.18	−.05, .33	−.05, .32
Interpersonal-Affective 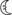 Involvement 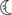 CU Traits	.03	.02	.22	−.01, .07	−.01, .07
Interpersonal-Affective 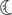 Conflict 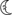 CU Traits	.06	.03	.03	.02, .12	.02, .11
Total Impulsive-Antisocial Traits	−.08	.09	.37	−.25, .09	−.24, .10
Impulsive-Antisocial 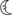 Involvement 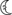 CU Traits	.00	.01	.92	−.01 .03	−.02, .02
Impulsive-Antisocial 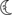 Conflict 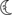 CU Traits	.01	.02	.48	−.02 .06	−.02, .06

Note.

1 = survived for multiple comparisons (.05/6 = *p* < .008).

CU = callous-unemotional. All models included parent-reported adolescent gender, race, age, two parent household status, and annual family income. Models also included both factors as predictors to account for their overlap. In a separate set of analyses, all models were also repeated without covariates and the pattern of results was the same. Conflict = scale of harsh parenting. Involvement = scale of warm parenting.
