# Supplementary material for: Associations between parental psychopathic traits, parenting, and adolescent callous-unemotional traits
Source: Res Child Adolesc Psychopathol. Author manuscript; Available in PMC 2022 Nov 1. (PMC8455443; doi:10.1007/s10802-021-00841-w)
Supplement: 1732839_Sup [file NIHMS1732839-supplement-1732839_Sup.docx]

**Supplemental Materials**

**Results**

**Are Associations Between Parenting and Adolescent CU Traits Explained by Non-Shared Environmental Influences, Accounting for the Overlap Between Parenting Dimensions?**

Within informant, when accounting for the overlap of parenting dimensions (i.e., whether there were unique effects of warmth versus harsh parenting on CU traits), there were significant associations between differences in harsh parenting (mother and father report) and differences in MZ twin CU traits (Supplemental Table 3). Specifically, based on mother report, when accounting for overlap, differences in mother harsh parenting were positively associated with twin differences in CU traits, whereas the association with differences in mother warmth was not significant. Similarly, there was a significant positive association between differences in father harsh parenting and differences in MZ twin CU traits, whereas the association with differences in warmth was not significant. There were no significant associations between differences in child-reported mother parenting practices and differences in child-reported MZ twin CU traits.

Across informant, there was one significant association between differences in mother-reported harsh parenting and differences in father-reported adolescent CU traits (Supplemental Table 3). Specifically, based on mother report of parenting, when accounting for their overlap, differences in mother harsh parenting were positively associated with differences in adolescent CU traits (father report of CU traits), whereas the association with warmth was not significant. There were no other significant associations between differences in parenting practices and differences in MZ twin CU traits across informant when accounting for the overlap between parenting dimensions.

**Does Twin Gender Moderate Associations?**

In a set of exploratory analyses, we also examined whether associations differed across boys and girls. First, regarding parental psychopathic traits and adolescent CU traits, two associations significantly differed across boys and girls within informant (father interpersonal-affective traits: Satorra-Bentler Scaled x^2^(1) = 8.52; *p*=.004, significant correcting for multiple comparisons; father impulsive-antisocial: Satorra-Bentler Scaled x^2^(1) = 5.82; *p*=.02, not significant correcting for multiple comparisons). Father interpersonal-affective traits were only related to higher adolescent CU traits in boys (B= .36; *p*<.001; df = 21, R^2^ = .15), but not in girls (B= .01; *p*=.97; df = 21, R^2^ = .02). The association between father impulsive-antisocial traits and adolescent CU traits was not significant in either boys or girls, but they differed in directionality (boys: B= .10, *p*=.30; df = 21, R^2^ = .12; girls: B= -.16, *p*=.11; df = 21, R^2^ = .04). Second, regarding parental psychopathic traits and parenting practices, one association significantly differed across boys and girls within informant (father impulsive-antisocial traits to father warmth: Satorra-Bentler Scaled x^2^(1) = 4.14; *p*=.04, not significant correcting for multiple comparisons). Father impulsive-antisocial traits were related to increased warmth at trend-level for girls (B= .19, *p*=.07; df = 27, R^2^ = .05), but not for boys (B= -.01, *p*=.93; df = 27, R^2^ = .14).

Third, in our models including psychopathic traits, parenting, and adolescent CU traits, there were three significant differences in pathways by twin gender for fathers: father interpersonal-affective traits to warmth (Satorra-Bentler Scaled x^2^(1) = 4.28; *p*=.04), father interpersonal-affective traits to adolescent CU traits (Satorra-Bentler Scaled x^2^(1) = 4.72; *p*=.03), and father impulsive98-antisocial traits to warmth (Satorra-Bentler Scaled x^2^(1) = 6.12; *p*=.01). Specifically, in boys, father interpersonal-affective traits were associated with reduced warmth (B= -.41, *p*=.001; df = 37, R^2^ = .16), whereas this was not significant in girls (B= -.07, *p*=.54; df = 37, R^2^ = .03). The association between interpersonal-affective traits and adolescent CU traits was not significant in either boys or girls, but they differed in directionality (B= .14, *p*=.11; df = 37, R^2^ = .34; B= -.09, *p*=.38; df = 37, R^2^ = .30, respectively). Additionally, in girls, father impulsive-antisocial traits were associated with increased warmth (B= .23, *p*= .02; df = 37, R^2^ = .05), whereas this association was not significant in boys (boys: B= -.02, *p*=.89; df = 37, R^2^ = .14). There was one significant difference in pathways by twin gender for mothers: mother interpersonal-affective traits to warmth (Satorra-Bentler Scaled x^2^(1) = 4.29; *p*=.04). However, the direction was the same for both boys (B= -.16, *p*=.02; df = 37, R^2^ = .12) and girls (B= -.35; *p*<.001; df = 37, R^2^ = .19). None of these associations survived correction for multiple comparisons.

Fourth, within informant, twin gender did not moderate associations between twin differences in parenting practices and twin differences in adolescent CU traits*.* Across informant, gender moderated the association between twin differences in child-reported mother warmth and twin differences in father-reported adolescent CU traits (β=.39, *p*=.02; df = 3; R^2^ = .20). Within boys, this association was negative and significant, whereas within girls this association was negative but not significant (Supplemental Table 4). This association did not survive correction for multiple comparisons.

Finally, within informant, twin gender did not moderate associations between twin differences in parenting practices and twin differences in adolescent CU traits, when accounting for the overlap of parenting dimensions*.* Across informant, there was only one significant interaction, when accounting for the overlap of parenting dimensions. Gender moderated the association between twin differences in child-reported mother warmth and twin differences in father-reported adolescent CU traits (β= .53, *p*= .02; df = 5, R^2^ = .21). Within boys, this association was negative and significant, whereas within girls this association was negative but not significant (Supplemental Table 4). This association did not survive correction for multiple comparisons.

| Supplemental Table 1  Zero-Order Correlations Between Parental Psychopathy and Dimensions of Parenting | | | | | | | | | |  |
| --- | --- | --- | --- | --- | --- | --- | --- | --- | --- | --- |
|  | Mother  Interpersonal-Affective | Mother Impulsive-Antisocial | Father Interpersonal-Affective | Father Impulsive-Antisocial | Mother Involvement (Mom Report) | Mother Conflict  (Mom Report) | Mother Involvement (Child Report) | Mother Conflict  (Child Report) | Father Involvement (Dad Report) | |
| Mother Impulsive-Antisocial Traits | .62*** |  |  |  |  |  |  |  |  | |
| Father Interpersonal-Affective Traits | .14** | -.01 |  |  |  |  |  |  |  | |
| Father Impulsive-Antisocial Traits | .11* | .06 | .73*** |  |  |  |  |  |  | |
| Mother Involvement  (Mom Report) | -.28*** | -.17*** | -.03 | -.05 |  |  |  |  |  | |
| Mother Conflict (Mom Report) | .28*** | .22*** | .13* | .03 | -.52*** |  |  |  |  | |
| Mother Involvement  (Child Report) | -.09^+^ | -.08 | -.06 | -.05 | .33*** | -.33*** |  |  |  | |
| Mother Conflict (Child Report) | .12* | .05 | .07 | .04 | -.25*** | .47*** | -.63*** |  |  | |
| Father Involvement  (Dad Report) | -.02 | .00 | -.19** | -.09 | .18*** | -.17** | .18** | -.14* |  | |
| Father Conflict  (Dad Report) | .08 | .06 | .32*** | .23*** | -.14* | .28*** | -.19** | .21*** | -.54*** | |
| Note. *p* < .10^+^, *p* < .05*, *p* < .01**, *p* < .001***. Involvement= scale of parental warmth. Conflict = scale of harsh parenting. | | | | | | | | | |  |

| Supplemental Table 2  Correlations Between Demographic Factors and Primary Study Variables | | | |
| --- | --- | --- | --- |
|  | Gender | Age | Income |
| Adolescent CU Traits (Mom Report) | -.14** | .19*** | -.06 |
| Adolescent CU Traits (Dad Report) | -.05 | .11* | -.05 |
| Adolescent CU Traits (Child Report) | -.27*** | .08^+^ | -.17*** |
| Mother Interpersonal-Affective Traits | .04 | .09* | -.08 |
| Mother Impulsive-Antisocial Traits | .00 | -.02 | -.17*** |
| Father Interpersonal-Affective Traits | -.11* | -.01 | -.04 |
| Father Impulsive-Antisocial Traits | -.12* | -.03 | -.17*** |
| Mother Involvement (Mom Report) | .08^+^ | -.27*** | .05 |
| Mother Conflict (Mom Report) | -.02 | .18*** | -.11* |
| Mother Involvement (Child Report) | .14** | -.12* | .14** |
| Mother Conflict (Child Report) | .03 | .20*** | -.15** |
| Father Involvement (Dad Report) | -.04 | -.15** | .02 |
| Father Conflict (Dad Report) | -.07 | -.09 | .05 |
| Note. **p<*.05, ***p<*.01, ****p<*.001. CU = callous-unemotional. Income= annual family income. Gender was coded as 0= Male, 1=Female. Involvement= scale of parental warmth. Conflict = scale of harsh parenting. | | | |

| Supplemental Table 3  Associations Between Monozygotic Difference Scores of Adolescent Callous-Unemotional Traits and Dimensions of Parenting, Controlling for the Overlap Between Involvement and Conflict | | | | | | | | | | | | | | | | | | | | | |  |  |
| --- | --- | --- | --- | --- | --- | --- | --- | --- | --- | --- | --- | --- | --- | --- | --- | --- | --- | --- | --- | --- | --- | --- | --- |
|  | Difference in Adolescent CU Traits  (Mom Report) | | | | | Difference in Adolescent CU Traits  (Dad Report) | | | | | | | | Difference in Adolescent CU Traits (Child Report) | | | | | | |  |  |  |
|  | B | SE | β | *p* | B | | | SE | | | β | | *p* | B | | SE β | | *p* | | |  |  |  |
| **Mothers** | | | | | | | | | | | | | | | | | | | | | |  |  |
| Difference in Mother Involvement  (Mom Report) | -.57 | .32 | -.19 | .08^+^ | | -.29 | | | .32 | | -.12 | .36 | | -.06 | .27 | | -.03 | | | .84 | | | |
| Difference in Mother Conflict  (Mom Report) | .50 | .21 | .26 | .02 | | | .49 | | | .18 | .36 | .007 | | .21 | .18 | | .14 | | | .25 | | | |
| df | 2 | | | | | | 2 | | | | | | |  |  | | 2 | | |  | | | |
| R^2^ | .15** | | | | .18** | | | | | | | | | .02 | | | | | | | | |  |
|  |  |  | |  |  | | |  | | |  | | |  |  | | | |  | | | |  |
| Difference in Mother Involvement  (Child Report) | -.37 | .23 | -.21 | .12 | | | -.25 | | | .21 | -.19 | .23 | | -.19 | .17 | | -.15 | | | .27 | | | |
| Difference in Mother Conflict  (Child Report) | -.01 | .22 | -.00 | .98 | | | .12 | | | .19 | .10 | .54 | | .22 | .16 | | .18 | | | .18 | | | |
| df |  | 2 | |  | | | 2 | | | | | | |  |  | | 2 | | |  | | | |
| R^2^ | .05 | | | | | | .07^+^ | | | | | | | .08* | | | | | | | | | |
| **Fathers** | | | | | | | | | | | | | | | | | | | | | |  |  |
| Difference in Father Involvement  (Dad Report) | -.10 | .46 | -.03 | .84 | | | .16 | | | .30 | .07 | .60 | | -.34 | .30 | | -.17 | | | .27 | | | |
| Difference in Father Conflict  (Dad Report) | .50 | .31 | .23 | .11 | | | .90 | | | .20 | .57 | <.001 | | -.02 | .21 | | -.02 | | | .92 | | | |
| df | 2 | | | | | | 2 | | | | | | | 2 | | | | | | | | | |
| R^2^ | .06 | | | | | | .29*** | | | | | | | .03 | | | | | | | | | |
| Note. ^+^*p*<.10, **p*<.05, ***p*<.01, ****p*<.001. CU = callous-unemotional. Df = degrees of freedom. Separate regression models were run to examine associations among parenting difference scores for each informant (including both conflict and involvement in the same model) predicting child CU traits difference scores for each informant (9 models total). Models included both dimensions of parenting to control for their overlap. There were 109 monozygotic twin pairs out of 275 total twin pairs. Involvement= scale of parental warmth. Conflict = scale of harsh parenting. | | | | | | | | | | | | | | | | | | | | | |  |  |

| Supplemental Table 4  Correlations Between Monozygotic Twin Difference Scores of Adolescent Callous-Unemotional Traits and Dimensions of Parenting Split by Gender | | | | |
| --- | --- | --- | --- | --- |
| Boys |  |  |  |  |
|  | Adolescent CU Traits  (Mom Report) | Adolescent CU Traits (Dad Report) | Adolescent CU Traits  (Child Report) |  |
| Mother Involvement  (Mom Report) | -.42** | -.35* | -.09 |  |
| Mother Conflict  (Mom Report) | .48*** | .53** | .15 |  |
| Mother Involvement  (Child Report) | -.33* | -.60*** | -.21 |  |
| Mother Conflict  (Child Report) | .16 | .40* | .30* |  |
| Father Involvement  (Dad Report) | .14 | -.10 | .01 |  |
| Father Conflict  (Dad Report) | .04 | .64*** | -.09 |  |
| Girls |  |  |  |  |
|  | Adolescent CU Traits  (Mom Report) | Adolescent CU Traits (Dad Report) | Adolescent CU Traits  (Child Report) |  |
| Mother Involvement  (Mom Report) | -.24 | -.30^+^ | -.11 |  |
| Mother Conflict  (Mom Report) | .25 | .38* | .17 |  |
| Mother Involvement  (Child Report) | -.11 | -.01 | -.33* |  |
| Mother Conflict  (Child Report) | .14 | .08 | .19 |  |
| Father Involvement  (Dad Report) | -.36* | -.32^+^ | -.28 |  |
| Father Conflict  (Dad Report) | .40* | .44* | .18 |  |
| Note. p < .10^+^, p < .05*, p < .01**, p < .001***. CU = callous-unemotional. There were 109 monozygotic twin pairs (44 female, 65 male) out of 275 total twin pairs. Involvement= scale of parental warmth. Conflict = scale of harsh parenting. | | | | |

**Supplemental Fig 1.** Example regression models of associations among parental psychopathic traits, parenting, and adolescent callous-unemotional traits. All models include child gender, race, age, two parent household status, and annual family income. 1A. Parental interpersonal-affective traits and parental impulsive-antisocial traits are predictors of adolescent callous-unemotional traits. A “within-informant” model, such that the same informant reports on each construct within the model (i.e., mother reports on both her own psychopathic traits and the child’s callous-unemotional traits). 1B. Parental interpersonal-affective traits and parental impulsive-antisocial traits are predictors of adolescent callous-unemotional traits. An “across-informant” model, such that there are unique reporters for different constructs within the model (i.e., mother reports her own psychopathic traits, dad reports on child’s callous-unemotional traits). 1C. Parental interpersonal-affective traits and parental impulsive-antisocial traits are predictors of parental involvement (warm/involved parenting) and parental conflict (harsh parenting). A “within-informant” model, such that the same informant reports on each construct within the model (i.e., mother reports on both her own psychopathic traits and her own parenting). 1D. Parental interpersonal-affective traits and parental impulsive-antisocial traits are predictors of parental involvement and parental conflict. An “across-informant” model, such that there are unique reporters for different constructs within the model (i.e., mother reports her own psychopathic traits, child reports on mother parenting). Involvement= scale of parental warmth. Conflict = scale of harsh parenting.

**
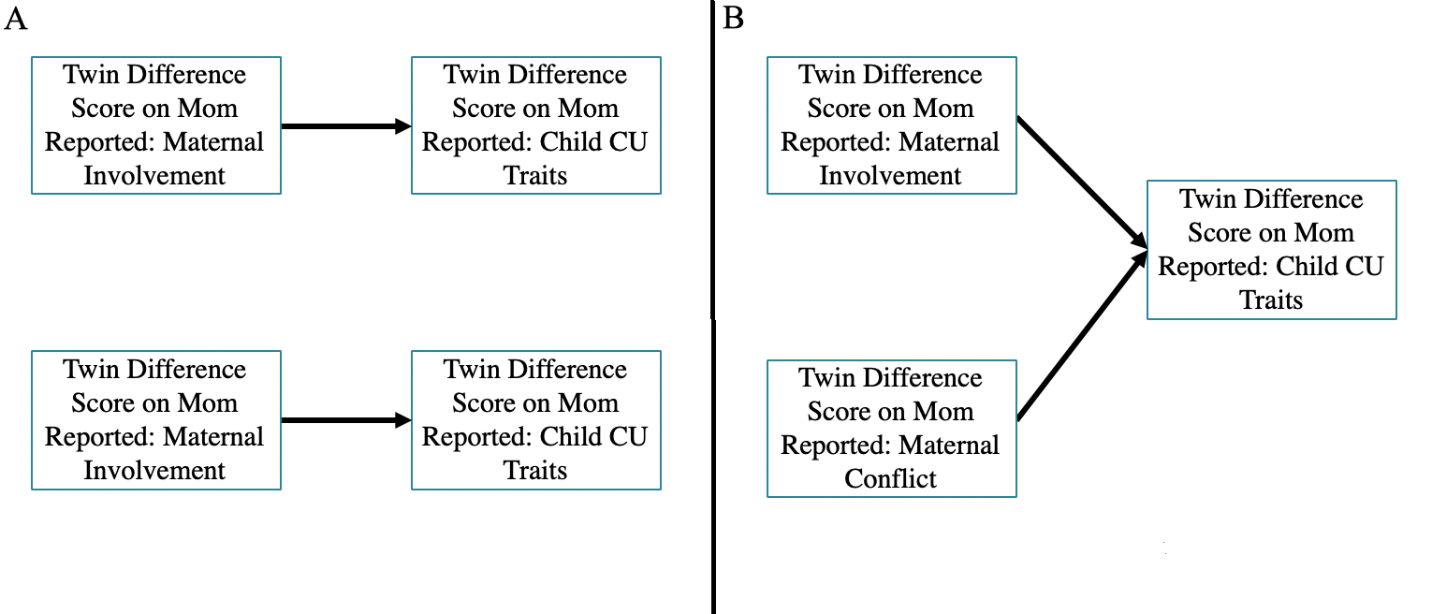
**

**Supplemental Fig 2.** Example models of associations between monozygotic difference scores in parenting and adolescent callous-unemotional traits. Figure 2A demonstrates zero-order correlations between monozygotic differences in one dimension of parenting (i.e., involvement or conflict) and adolescent callous-unemotional traits. Figure 2B demonstrates partial correlations between monozygotic differences in both dimensions of parenting and adolescent callous-unemotional traits, controlling for the overlap between parenting dimensions. Involvement= scale of parental warmth. Conflict = scale of harsh parenting.
